# Heterogeneous effects of preoperative beta blockers on 30-day mortality after coronary artery bypass surgery

**DOI:** 10.1080/07853890.2026.2719256

**Published:** 2026-09-04

**Authors:** Wenyuan Lu, Hanwei Tang, Jingyu Wang, Jianing Han, Yuanming Li, Xin Wang, Jianfeng Hou

**Affiliations:** a Cardiac Surgery Centre, Fuwai Hospital, National Center for Cardiovascular Diseases, Chinese Academy of Medical Sciences and Peking Union Medical College, Beijing, China; b Department of Epidemiology, Fuwai Hospital, National Center for Cardiovascular Diseases, Chinese Academy of Medical Sciences and Peking Union Medical College, Beijing, China

**Keywords:** Iterative causal forest, coronary artery bypass surgery, β-blockers, heterogeneous treatment effect

## Abstract

**Background:**

The role of preoperative β-blocker therapy in patients with ventricular dysfunction undergoing coronary artery bypass grafting (CABG) remains unclear.

**Objective:**

We aim to evaluate heterogeneous treatment effects of preoperative β-blocker therapy in patients with ventricular dysfunction undergoing CABG and to identify subgroups that may derive greater benefit from preoperative β-blocker therapy than untreated controls.

**Methods:**

To investigate the heterogeneous treatment effects of preoperative β-blocker therapy on 30-day mortality in patients with ventricular dysfunction undergoing CABG, we analyzed data on 6,492 patients in the Chinese Cardiac Surgery Registry database between 2017 and 2020. After propensity score matching, we applied a machine-learning iterative causal forest (iCF) algorithm to estimate individualized treatment effects (ITEs) of β-blockers on 30-day all-cause mortality after CABG.

**Results:**

The iCF models showed heterogeneity in the effects of preoperative β-blockers on 30-day mortality. Estimated glomerular filtration rate (eGFR), left ventricular end-diastolic diameter (LVEDD) and body mass index (BMI) were identified by the algorithm to distinguish patients with heterogeneous treatment effects. Among patients with eGFR 66 mL/min/1.73 m^2^ or less, preoperative β-blocker therapy was associated with a significantly lower risk of death from any cause (adjusted odds ratio [aOR], 0.39; 95% CI, 0.22 to 0.67; *p* = 0.001). No significant benefits were found for other subgroups.

**Conclusion:**

Machine learning analysis revealed treatment effect heterogeneity, with preoperative β-blockers demonstrating superior outcomes specifically in CABG patients with ventricular dysfunction and concomitant renal dysfunction (eGFR ≤ 66 mL/min/1.73 m^2^).

## KEY MESSAGES

1. Preoperative β-blocker therapy was associated with heterogeneous 30-day mortality outcomes among patients with ventricular dysfunction undergoing CABG.
2. The iCF algorithm identified eGFR, LVEDD, and BMI as potential treatment-effect modifiers, with a lower 30-day mortality observed among patients with an eGFR of 66 mL/min/1.73 m^2^ or less.

## 1. Introduction

Myocardial revascularization by coronary artery bypass grafting (CABG) has been the most durable and complete treatment of patients with coronary artery disease (CAD) [1]. Medication therapy is key to achieving optimal perioperative and long-term success in cardiac surgery. A substantial proportion of patients undergoing cardiac surgery are currently receiving β-blocker therapy [2,3]. Some studies demonstrate that β-blocker therapy should be maintained in both elective and emergency cardiac surgery to reduce mortality ^[^4] and postoperative arrhythmias [5,6]. However, other studies suggested no significant mortality benefit with preoperative β-blockers in CABG patients [7,8]. A recent meta-analysis further demonstrated that preoperative β-blocker therapy did not reduce operative mortality or the incidence of postoperative complications [9]. Additionally, maintaining β-blocker therapy until surgery in chronic users could impair the effectiveness of catecholamines and increase the risk of bradycardia and hypotension in the early postoperative period [10,11]. Whether one should initiate β-blockers in the preoperative period is less clear [12].

Selected patients undergoing CABG may benefit from preoperative β-blocker therapy. It is suggested that the benefit of β-blockers before CABG to prevent myocardial infarction (MI) and death is limited only to patients with a recent MI [13]. However, the effectiveness and safety of preoperative β-blocker therapy in patients with left ventricular dysfunction undergoing CABG have not yet been adequately assessed. Some studies have suggested that, among patients with reduced left ventricular ejection fraction (LVEF) (<30%), the association between preoperative β-blocker therapy and 30-day mortality has been inconsistent across observational studies. Although no statistically significant difference was observed, the direction of effect estimates varied, suggesting that the perioperative effects of β-blockers may differ in patients with ventricular dysfunction [3,14]. Tang et al. demonstrated that preoperative β-blocker therapy in CABG was not associated with improved surgical mortality or reduced complications in patients with left ventricular dysfunction [15]. Such inconsistency may reflect differences in baseline ischemic burden, myocardial reserve, ventricular remodeling, renal function, and susceptibility to perioperative hemodynamic instability. These factors are particularly relevant in patients undergoing CABG with reduced LVEF, who often present with complex and heterogeneous clinical profiles that may modify their responses to preoperative β-blocker therapy and surgical revascularization. Therefore, it is crucial to identify the treatment effects for each individual or subgroup with similar demographic or physiologic features, which are called ‘heterogeneous treatment effect’ (HTE).

The causal forest analysis is a machine learning method designed to estimate causal effects, particularly useful for assessing heterogeneous effects of individuals [16]. Iterative causal forest (iCF) is a causal forest-based subgrouping algorithm, which pinpoints important subgroups with HTE without requiring prior knowledge of treatment effect modifiers and often outperforms other subgrouping methods [17,18]. Therefore, we applied iCF to examine treatment-effect heterogeneity among patients with ventricular dysfunction undergoing CABG to identify a certain subgroup that may derive greater benefit from preoperative β-blocker therapy with respect to 30-day mortality.

## 2. Methods

### 2.1. Study design and participants

We conducted the analysis based on the Chinese Cardiac Surgery Registry (CCSR) database from 2017 to 2020 [19]. The CCSR registry operates under the supervision of the steering committee composed of cardiac surgeons and researchers from Fuwai Hospital and the National Center for Cardiovascular Diseases (NCCD), containing information on CABG from 94 hospitals. Each participating hospital performs more than 100 cardiac surgeries per year and is required to record cases using a standardized case report form. We analyzed data from 6,492 patients in the CCSR with LVEF ≤50% and without preoperative intra-aortic balloon pump insertion, cardiogenic shock, or third-degree heart block.

The study was conducted in accordance with the Declaration of Helsinki and approved by the Institutional Review Board of Fuwai Hospital (protocol code 2017-887). The written informed consent was obtained from all participants.

### 2.2. Exposure definition

Preoperative β-blocker exposure was defined as documented administration of at least one dose of any β-blocker within 24 h before CABG.

### 2.3. Study outcome

The outcome was all-cause mortality at 30 days after CABG, which is a clinically established measure of perioperative mortality after cardiac surgery and is directly relevant to the short-term benefits and hemodynamic risks potentially associated with preoperative β-blocker therapy.

### 2.4. Other covariates

Candidate variables for the iCF model were selected based on previous studies, clinical expertise, and data availability in the registry. All clinically relevant preoperative and operative variables that were available in the dataset were considered. Variables collected include: age, body mass index (BMI), sex, diabetes mellitus (DM), hypertension, hyperlipidemia, chronic obstructive pulmonary disease (COPD), chronic renal failure (CRF), cerebrovascular accident, previous MI, previous percutaneous coronary intervention (PCI), Canadian Cardiovascular Society (CCS) classification, New York Heart Association (NYHA) classification, left main CAD, estimated glomerular filtration rate (eGFR), congestive heart failure (CHF), peripheral artery disease, symptomatic CAD, atrial fibrillation (AF), prior cardiovascular surgery, low-density lipoprotein cholesterol (LDL-c), left ventricular end-diastolic diameter (LVEDD), left atrial diameter (LAD), carotid disease, aortic regurgitation (AR), mitral regurgitation (MR), tricuspid regurgitation (TR), cardiopulmonary bypass (CPB), triple vessel disease, using radial artery and use of angiotensin-converting enzyme inhibitor (ACEI). Missing covariates were imputed using a random forest algorithm. To reduce redundancy and potential multicollinearity, pairwise Pearson correlation analysis was performed among candidate variables before model construction.

### 2.5. Propensity score matching

We performed 1:1 optimal propensity score matching based on the propensity score to balance the characteristics between participants who used β-blockers preoperatively and those who did not. The propensity score was estimated using a logistic regression based on several potential confounding factors including symptomatic CAD, CHF, previous MI, previous PCI, NYHA, CCS classification, hypertension, AF, prior cardiovascular surgery, left main CAD and triple vessel disease. This matching approach resulted in 2,489 matched pairs, ensuring balanced comparability between the groups. The absolute standardized mean difference <0.1 indicated successful balance [20].

### 2.6. Statistical analysis

Continuous variables were presented as mean (SD) and compared by Student t test. Categorical variables were summarized as frequencies with percentages, and group differences were assessed using chi-square tests.

We used the iCF approach to identify subgroups of patients undergoing CABG who may derive differential benefit from preoperative β-blocker therapy. The analysis followed a causal-inference framework. First, machine-learning models were used to estimate the outcome and the propensity score based on baseline covariates, and residualized outcomes were created to reduce confounding. A pilot causal forest was then fitted to assess treatment-effect heterogeneity and to rank potential effect modifiers. Variables with above-average importance were retained for subgroup discovery. To obtain stable and interpretable subgroups, multiple shallow causal forests with varying tree depths were repeatedly trained. In accordance with the iCF implementation, minimum leaf size was tuned for candidate subgroup trees with depths 2, 3, 4, and 5 as the main hyperparameter-tuning step. For each depth, the most frequently occurring tree structure across iterations was selected, and a synthetic decision tree was constructed. The final subgroup structure was chosen based on the lowest prediction error from 10-fold cross-validation. Within each identified subgroup, the treatment effect of preoperative β-blocker therapy on 30-day mortality was estimated as an adjusted risk difference (aRD). These aRDs were derived using stabilized inverse-probability weighting, which created weighted treated and untreated groups with comparable baseline characteristics. The difference in weighted risks between the two treatment arms represented the subgroup-specific causal effect [21]. The implementation of subgroup identification was performed using the package iCF. *p* < 0.05 was considered to be statistically significant. All statistical analyses were performed in R software (version 4.5.1).

### 2.7. Sensitivity analyses

To assess the robustness of the primary findings, we performed several sensitivity analyses: (1) bootstrap validation using 1,000 resamples drawn from the propensity score-matched cohort; (2) replication of the analysis in the complete-case unmatched cohort after excluding patients with missing values for the three iCF-identified variables (*n* = 4,751); (3) additional adjustment for six major postoperative complications (prolonged mechanical ventilation, reintubation, reoperation, postoperative myocardial infarction, postoperative stroke, and postoperative renal failure); and (4) a mixed-effects logistic regression analysis with hospital included as a random intercept to account for center-level heterogeneity.

## 3. Results

### 3.1. Study participants

Figure S1 shows the flowchart of patient selection and study process. Among the 4,978 patients after propensity score matching, 2,489 received β-blockers preoperatively (mean age, 61 years; female patients, 370 (14.9%); mean BMI, 25.27 kg/m^2^, mean eGFR, 102.42 mL/min/1.73 m^2^; mean LVEDD, 60.07 mm) while another 2,489 patients did not receive β-blockers prior to surgery (mean age, 61 years; female patients, 427 (17.2%); mean eGFR, 93.94 mL/min/1.73 m^2^; mean LVEDD, 57.51 mm). The use of β-blockers was not associated with improved outcomes in the matched cohort (Table 1). The correlation heatmap showed generally low pairwise correlations among candidate variables, with no evidence of substantial redundancy or severe multicollinearity (Figure S2).

**Table 1.** Baseline demographic and clinical characteristics in overall cohort.

	β-blockers use groups		*p* value
	No (*n* = 2,489)	Yes (*n* = 2,489)	
Death	110	83	0.056
Baseline characteristics			
Age, mean (SD)	61.42 (9.35)	60.86 (9.25)	0.034
Female, No. (%)	427 (17.2)	370 (14.9)	0.030
BMI, mean (SD)	24.67 (3.11)	25.27 (3.33)	<0.001
DM, No. (%)	799 (32.1)	877 (35.2)	0.021
Hypertension, No. (%)	1308 (52.6)	1339 (53.8)	0.394
Hyperlipidaemia, No. (%)	855 (34.4)	839 (33.7)	0.654
CRF, No. (%)	45 (1.8)	51 (2.0)	0.606
Peripheral artery disease, No. (%)	114 (4.6)	111 (4.5)	0.891
Previous stroke, No. (%)	208 (8.4)	221 (8.9)	0.544
Symptomatic CAD, No. (%)	1984 (79.7)	2005 (80.6)	0.477
CHF, No. (%)	136 (5.5)	154 (6.2)	0.304
CCS^a^, No. (%)	1604 (64.4)	1588 (63.8)	0.658
NYHA^b^, No. (%)	1327 (53.3)	1370 (55.0)	0.232
AF, No. (%)	56 (2.2)	55 (2.2)	1.000
Previous MI, No. (%)	1039 (41.7)	1060 (42.6)	0.566
Previous PCI, No. (%)	278 (11.2)	285 (11.5)	0.788
Prior cardiovascular surgery, No. (%)	54 (2.2)	45 (1.8)	0.417
eGFR, mean (SD)	93.94 (80.83)	102.42 (338.17)	0.224
LDL-c, mean (SD)	2.37 (1.04)	2.87 (12.53)	0.050
Left main CAD, No. (%)	702 (28.2)	764 (30.7)	0.058
Triple vessel disease, No. (%)	1777 (71.4)	1791 (72.0)	0.683
LVEDD, mean (SD)	57.51 (8.49)	60.07 (136.02)	0.349
LAD, mean (SD)	39.81 (5.80)	39.90 (6.38)	0.585
AR, No. (%)	27 (1.1)	45 (1.8)	0.044
MR, No. (%)	284 (11.4)	272 (10.9)	0.621
TR, No. (%)	56 (2.2)	53 (2.1)	0.846
Carotid disease, No. (%)	466 (18.7)	352 (14.1)	<0.001
CPB, No. (%)	1434 (57.6)	1531 (61.5)	0.006
Radial artery, No. (%)	69 (2.8)	88 (3.5)	0.144
ACEI, No. (%)	533 (21.4)	904 (36.3)	<0.001

Note: Continuous and categorical variables are presented as mean (SD) and number (%), respectively.

Abbreviations: BMI: body mass index; DM: diabetes mellitus; CRF: chronic renal failure; CAD: coronary artery disease; CHF: congestive heart failure; CCS: Canadian Cardiovascular Society; NYHA: New York Heart Association; AF: atrial fibrillation; MI: myocardial infarction; PCI: percutaneous coronary intervention; eGFR: estimated glomerular filtration rate; LDL-c: low-density lipoprotein cholesterol; LVEDD: left ventricular end-diastolic diameter; LAD: left atrial diameter; AR: aortic regurgitation; MR: mitral regurgitation; TR: tricuspid regurgitation; CPB: cardiopulmonary bypass; ACEI: angiotensin-converting enzyme inhibitor; SD: standard deviation.

### 3.2. Development of the iCF models

**Figure 1.**
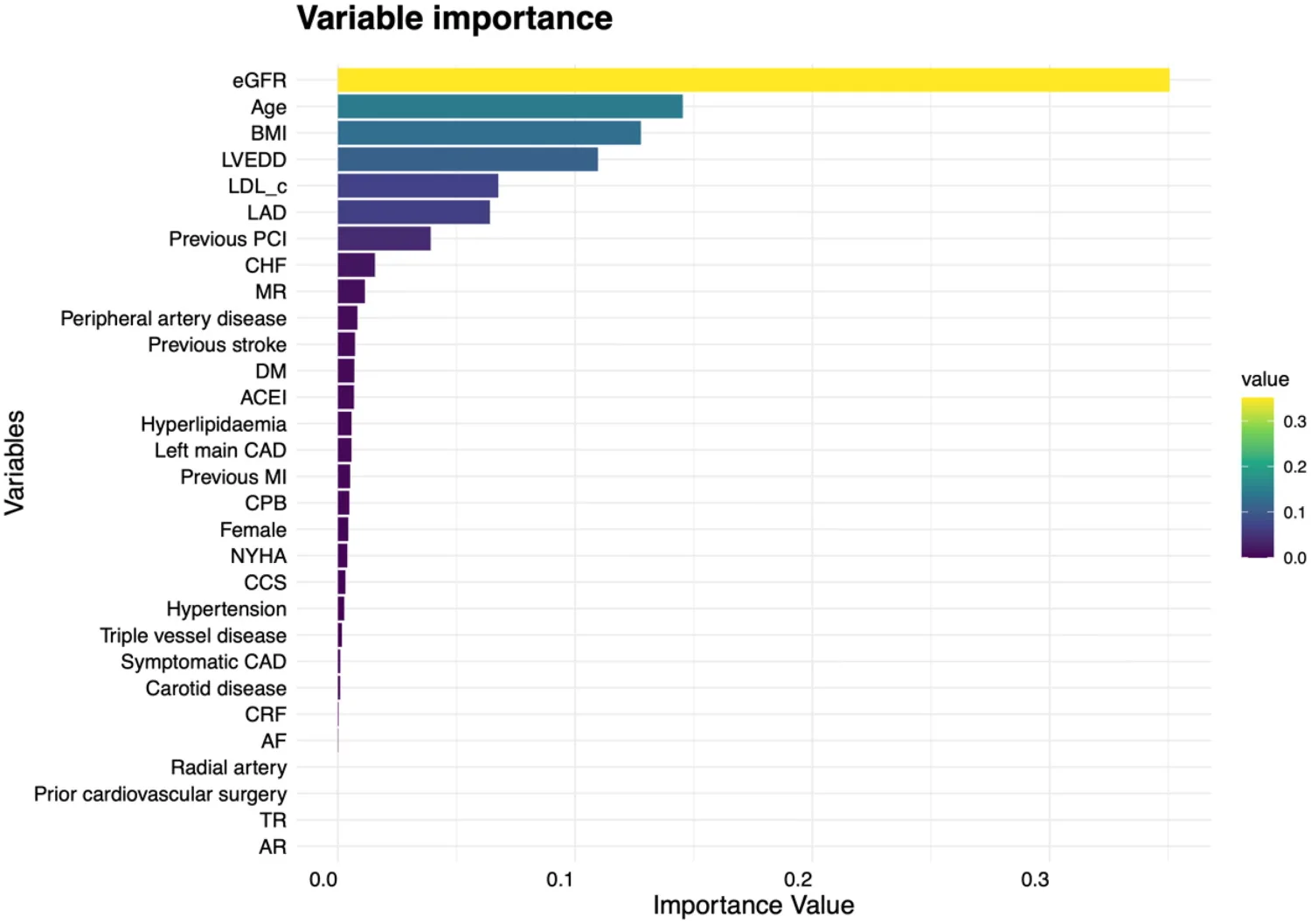
Variable importance measures of the clinical variables included in the iCF algorithm. Abbreviations: eGFR: estimated glomerular filtration rate; BMI: body mass index; LVEDD: left ventricular end-diastolic diameter; LDL-c: low-density lipoprotein cholesterol; LAD: left atrial diameter; PCI: percutaneous transluminal coronary intervention; CHF: congestive heart failure; MR: mitral regurgitation; DM: diabetes mellitus; ACEI: angiotensin-converting enzyme inhibitor; CAD: coronary artery disease; CPB: cardiopulmonary bypass; NYHA: New York Heart Association; CCS: Canadian Cardiovascular Society; CRF: chronic renal failure; AF: atrial fibrillation; TR: tricuspid regurgitation; AR: aortic regurgitation.

**Figure 2.**
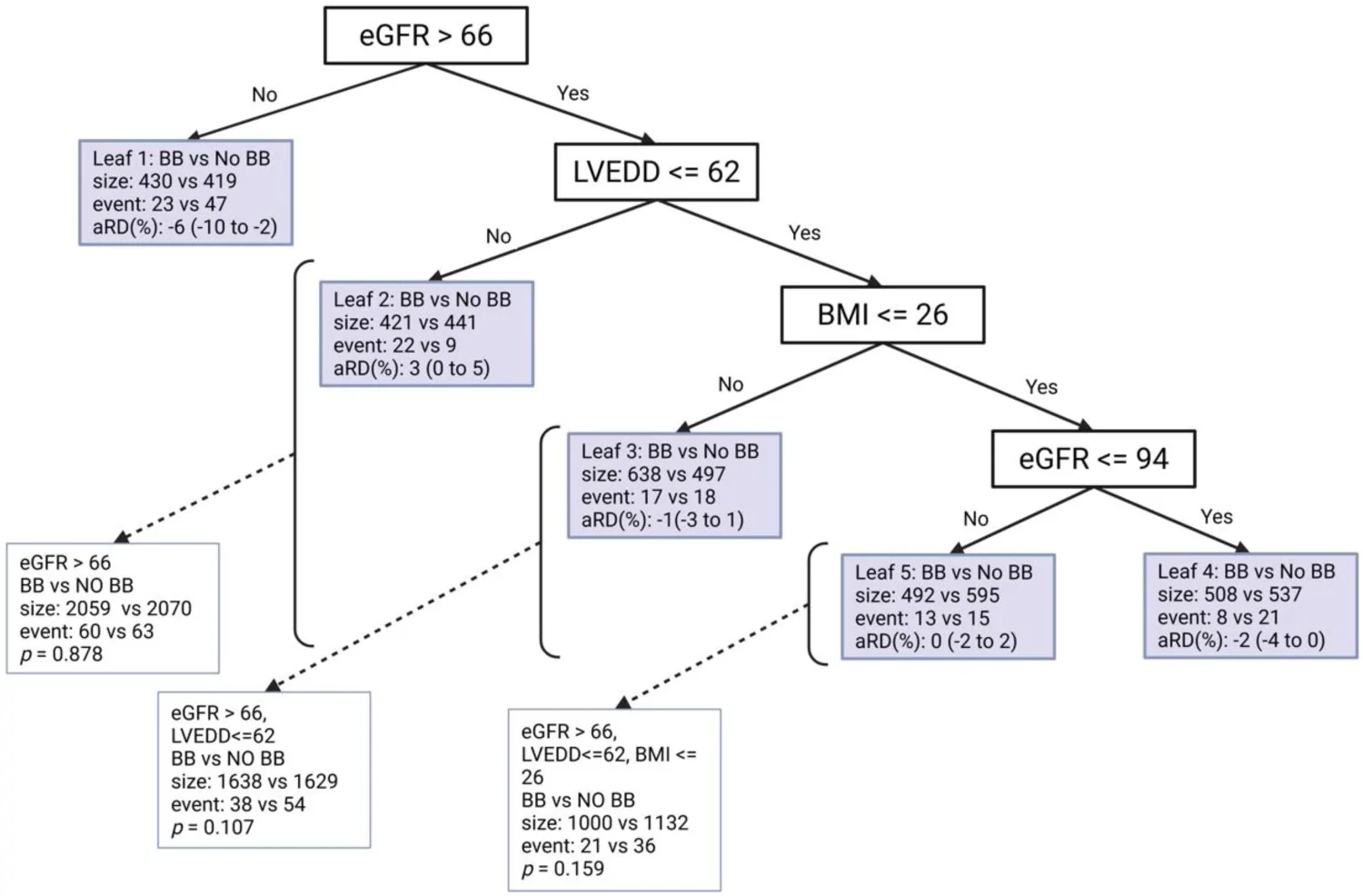
The iCF algorithm identified 5 subgroups with HTEs. Abbreviations: BB: beta-blockers; aRD: adjusted risk difference.

We observed heterogeneity in the effects of preoperative β-blocker therapy on 30-day mortality. Variable importance measures from the iCF analysis are shown in Figure 1. The algorithm identified three variables (eGFR, LVEDD, and BMI) as key variables distinguishing subgroups with different estimated treatment effects. On the basis of the identified variables, the iCF model divided individuals into five subgroups with varied risk differences, as seen in Figure 2. Subgroup 1: eGFR ≤ 66 mL/min/1.73 m^2^; Subgroup 2: eGFR > 66 mL/min/1.73 m^2^ and LVEDD > 62 mm; Subgroup 3: eGFR > 66 mL/min/1.73 m^2^, LVEDD ≤ 62 mm and BMI > 26 kg/m^2^; Subgroup 4: eGFR > 66 mL/min/1.73 m^2^, LVEDD ≤ 62 mm, BMI ≤ 26 kg/m^2^ and eGFR ≤ 94 mL/min/1.73 m^2^; Subgroup 5: eGFR > 66 mL/min/1.73 m^2^, LVEDD ≤ 62 mm, BMI ≤ 26 kg/m^2^ and eGFR > 94 mL/min/1.73 m^2^.

### 3.3. Heterogeneous treatment effects between the subgroups

Specifically, subgroup 1 consisted of 849 patients (17.1% of the overall study population) and was characterized by eGFR 66 mL/min/1.73 m^2^ or less. The patients in subgroup 1 receiving preoperative β-blockers or not showed the significant difference on 30-day mortality between the two groups (*p* = 0.001) (Table 2). The aRD was −6% (95% CI, −10% to −2%) and the adjusted risk reduction was 61% (adjusted odds ratio [aOR], 0.39; 95% CI, 0.22 to 0.67; *p* = 0.001) by preoperative β-blockers in this subgroup.

**Table 2.** Associations between preoperative β-blocker therapy and 30-day all-cause mortality within the subgroups identified by the iCF analysis.

Subgroups	No. of events/total		Unadjusted		Adjusted		
	No β-blockers	β-blockers	OR (95% CI)	*p*	OR (95% CI)	*p*	Bonferroni-adjusted *p*
Subgroup 1	47/419	23/430	0.45 (0.26, 0.74)	0.002	0.39 (0.22, 0.67)	0.001	0.005
Subgroup 2	9/441	22/421	2.65 (1.24, 6.12)	0.015	2.27 (1.03, 5.40)	0.050	0.250
Subgroup 3	18/497	17/638	0.73 (0.37, 1.43)	0.357	0.75 (0.37, 1.54)	0.437	1.000
Subgroup 4	21/537	8/508	0.39 (0.16, 0.86)	0.026	0.48 (0.19, 1.06)	0.083	0.415
Subgroup 5	15/595	13/492	1.05 (0.49, 2.23)	0.900	0.85 (0.37, 1.91)	0.701	1.000

Note: Adjusted for symptomatic CAD, previous MI, previous PCI, CCS, hypertension, ACEI and age. Subgroup 1: eGFR ≤ 66 mL/min/1.73 m^2^; Subgroup 2: eGFR > 66 mL/min/1.73 m^2^ and LVEDD > 62 mm; Subgroup 3: eGFR > 66 mL/min/1.73 m^2^, LVEDD ≤ 62 mm and BMI > 26 kg/m^2^; Subgroup 4: eGFR > 66 mL/min/1.73 m^2^, LVEDD ≤ 62 mm, BMI ≤ 26 kg/m^2^ and eGFR ≤ 94 mL/min/1.73 m^2^; Subgroup 5: eGFR > 66 mL/min/1.73 m^2^, LVEDD ≤ 62 mm, BMI ≤ 26 kg/m^2^ and eGFR > 94 mL/min/1.73 m^2^. Bonferroni-adjusted p values were calculated based on the adjusted model p values across the five iCF-defined subgroup comparisons.

A similar trend was observed in Subgroup 3 (22.8% of the overall study population) and Subgroup 4 (21.0% of the overall study population). Preoperative β-blocker therapy was associated with numerically lower 30-day mortality in these two subgroups, with aRDs of −1% (95% CI, −3% to 1%) and −2% (95% CI, −4% to 0), and aORs of 0.75 (95% CI, 0.37 to 1.54; *p* = 0.437) and 0.48 (95% CI, 0.19 to 1.06; *p* = 0.083), respectively. In Subgroup 2 (17.3% of the overall study population), preoperative β-blocker therapy showed a nominal association with increased 30-day mortality (aRD, 3%; 95% CI, 0 to 5%; aOR, 2.27; 95% CI, 1.03 to 5.40; *p* = 0.050). In Subgroup 5 (21.8% of the overall study population), there was no significant difference in 30-day mortality between patients who received preoperative β-blockers and those who did not, with a p-value of 0.701 and aOR of 0.85 (95% CI, 0.37 to 1.91; *p* = 0.701).

### 3.4. Sensitivity analyses

We generated 1,000 bootstrap samples from the propensity score-matched cohort and reassessed the associations between preoperative β-blocker therapy and 30-day mortality across all iCF-defined subgroups (Table S1). In Subgroup 1 (eGFR ≤ 66 mL/min/1.73 m^2^), the association remained robust, with a median adjusted OR of 0.38 (95% CI, 0.20 to 0.68); 92.1% of bootstrap samples showed a statistically significant association. In contrast, the proportions of significant adjusted associations were lower in the remaining subgroups (47.4% in Subgroup 2 and ≤ 12.5% in Subgroups 3–5), supporting the robustness of the favorable association primarily observed in the low-eGFR subgroup. We excluded 1,741 patients with any missing values of the variables identified by iCF, repeated the analysis on the remaining 4,751 patients and reassessed the association between the preoperative β-blocker therapy and 30-day mortality utilizing the same causal tree-based subgrouping. A consistent finding was observed in Subgroup 1 (eGFR ≤ 66 mL/min/1.73 m^2^). Compared with patients who did not receive β-blockers, those with preoperative β-blocker therapy had a significantly lower risk of perioperative mortality (OR, 0.53; 95% CI, 0.31 to 0.91; *p* = 0.020). No association between β-blocker therapy and perioperative mortality was observed in any other subgroups except Subgroup 1 in the sensitivity analysis cohort (Table S2).

To further address potential confounding from postoperative clinical events, we additionally adjusted six major postoperative complications when evaluating the association between preoperative β-blocker therapy and lower 30-day mortality in the eGFR ≤66 mL/min/1.73 m^2^ subgroup, yielding results similar to those of the primary analysis (OR, 0.33; 95% CI, 0.17 to 0.65; *p* = 0.001) (Table S3). We also performed a mixed-effects logistic regression analysis with hospital included as a random intercept to account for potential center-level heterogeneity. Preoperative β-blocker therapy remained significantly associated with lower 30-day mortality in the eGFR ≤ 66 mL/min/1.73 m^2^ subgroup (OR, 0.53; 95% CI, 0.30 to 0.93; *p* = 0.028), consistent with the primary analysis (Table S4).

## 4. Discussion

Although previous studies did not find benefit on 30-day mortality from the preoperative β-blocker therapy in patients undergoing CABG, we demonstrated the HTEs of the effects of preoperative β-blocker therapy based on the iCF algorithm. The study population was divided into five subgroups with different risk-benefit profiles using three baseline variables including eGFR, LVEDD and BMI. Specifically, patients with an eGFR ≤66 mL/min/1.73 m^2^ appeared most likely to derive greater benefit from preoperative β-blocker therapy. These results emphasize the obscured HTEs of preoperative β-blocker therapy and might help to facilitate individualized clinical decision-making for identifying CABG patients who are optimal candidates for preoperative β-blocker therapy.

In evidence-based medicine, randomized controlled trials are regarded as the gold standard for clinical evidence, offering the strongest support for therapeutic decision-making. Existing prospective trials demonstrated that preoperative β-blockers provide clinical benefits for certain patient populations, though not without potential harms. Substantial person-level heterogeneity in treatment effects is commonly observed, indicating that the same treatment may have different effects across individuals because of biological and physiological variation. Thus, clinicians must carefully weigh these risks and benefits for each patient. Therefore, it is important to leverage routinely available clinical features to identify HTEs, provide more patient-centered evidence, and facilitate the interpretation of precision medicine in patients undergoing CABG. Subgroup analyses are the most common approach to identify characteristics that might be associated with HTEs. Nevertheless, conventional subgroup analyses were constrained by inadequate power, pre-existing assumptions and evaluating only one effect modifier at a time, providing very limited information for estimating how the effects of intervention vary across individuals [22,23]. Additionally, subgroup analyses are prone to false-positive results because of multiple comparisons and false-negative results related to inadequate test power [24]. Causal forest-based approaches can capture combinations of baseline characteristics and data-driven split points to characterize variation in treatment effects across subgroups, as demonstrated in other clinical settings [25]. In the present study, the iCF algorithm further evaluated candidate subgroup structures across increasing tree depths and selected the structure with the lowest cross-validated prediction error, thereby prioritizing stable HTE signals and reducing the likelihood of spurious subgroup findings [17].

Our analysis identified patients with eGFR ≤ 66 mL/min/1.73 m^2^ as deriving the greatest clinical benefit from the treatment. In clinical practice, renal impairment often poses a barrier to the initiation and up-titration of guideline-recommended therapies for heart failure, leading to concerns by clinicians about efficacy and safety [26]. However, the study by Kotecha et al. demonstrated that patients with heart failure and LVEF < 50% should receive β-blocker therapy even if they have moderate or moderately severe renal insufficiency [26]. Subgroup analysis from the MERIT-HF trial [27], as well as the CIBIS-II [28] and SENIORS trials [29] also suggested potential benefit from β-blockers in those with eGFR < 45 mL/min/1.73 m^2^. Moreover, the study by Molnar et al. provided further evidence that β-blocker therapy reduces all-cause mortality in elderly patients with congestive heart failure and chronic kidney disease who have an eGFR < 30 mL/min/1.73m^2 [^30]. In addition, we noted a higher prevalence of ACEI use among patients receiving preoperative β-blocker therapy. This difference may reflect variations in heart failure management or broader use of guideline-directed medical therapy. After adjustment for ACEI use, the association between preoperative β-blocker therapy and lower 30-day mortality in the low-eGFR subgroup remained significant and became more pronounced, suggesting that this finding was not explained by the observed imbalance in ACEI use.

While we observed that preoperative β-blocker therapy showed a trend toward increased perioperative mortality in selected CABG patients (eGFR > 66 mL/min/1.73 m^2^, LVEDD > 62 mm), this association was not statistically significant. In the bootstrap analysis, 47.4% of the 1,000 resamples showed a statistically significant association in the same direction, providing limited support for this potential signal. The study by Patlolla et al. confirmed that in patients with ischemic cardiomyopathy (preoperative left ventricular ejection fraction ≤35%) undergoing isolated CABG, a larger preoperative LVEDD is a significant risk factor for impaired left ventricular ejection fraction outcomes [31]. Steffen et al. also demonstrated that in patients with heart failure with mildly reduced ejection fraction, left ventricular dilation was associated with a higher risk of heart failure-related rehospitalization [32]. While direct clinical evidence is lacking regarding LVEDD as an effect modifier for β-blockers, the association of increased LVEDD with eccentric remodeling, elevated wall stress, systolic dysfunction, and neurohormonal activation [33] suggests its potential to amplify the perioperative adverse effects of these drugs. In addition, although some patients had relatively preserved eGFR, normal or higher eGFR does not necessarily indicate adequate renal reserve [34]. In a cohort of 8,189 patients with coronary artery disease undergoing coronary angiography, enlarged LVEDD was independently associated with an increased risk of contrast-associated acute kidney injury [35], suggesting greater renal vulnerability and reduced tolerance to hemodynamic instability. In this context, the negative chronotropic and negative inotropic effects of β-blockers may increase susceptibility to bradycardia, hypotension, reduced cardiac output, and impaired renal perfusion. These mechanisms may partly explain the trend observed in Subgroup 2. While eGFR significantly influences preoperative β-blocker utilization, its use should be guided by comprehensive assessment rather than eGFR alone. Moreover, given the high prevalence of CAD in chronic kidney disease (CKD) patients, future research should prioritize CKD-specific analyses [36].

This study has several limitations. First, the dataset did not capture detailed information regarding the specific type of β-blockers, dosage, timing of initiation, or duration of preoperative therapy. Different β-blockers, such as metoprolol, bisoprolol, and carvedilol, have distinct pharmacological profiles and may exert different effects on perioperative hemodynamics and clinical outcomes. The lack of detailed medication data limited our ability to explore whether the observed heterogeneous treatment effects varied by β-blocker type, dose, timing, or treatment duration. In addition, detailed intraoperative variables, including vasoactive drug use and hemodynamic management parameters, as well as information on other guideline-directed medical therapies, such as ARBs, mineralocorticoid receptor antagonists, and SGLT2 inhibitors, were unavailable in the registry. Future studies with more detailed medication information are needed to validate and extend our findings. Second, all analyses were limited to short-term outcomes. Any potential long-term benefits could not be evaluated. Finally, the eGFR threshold was generated by the iCF algorithm from the present dataset. Therefore, this data-driven cut-off should be interpreted as hypothesis-generating and requires further validation in independent CABG cohorts before being considered for clinical decision-making. Despite these limitations, sensitivity analyses confirmed the robustness of our results. By identifying stable HTEs, our study contributes to personalized patient decision-making and can inform the design of future research on preoperative β-blocker therapy. Prospective studies could first validate the data-driven eGFR threshold identified in our study in independent populations of patients with left ventricular dysfunction undergoing CABG. Future randomized trials could incorporate prespecified stratification by renal function to determine whether patients with renal impairment derive greater benefit from preoperative β-blocker therapy, while systematically evaluating perioperative hemodynamic and renal safety.

## 5. Conclusion

Using the iCF method, we identified significant heterogeneity in the effects of preoperative β-blocker therapy on perioperative mortality among CABG patients with ventricular dysfunction. Our machine-learning algorithm pinpointed three key treatment effect modifiers: eGFR, LVEDD, and BMI. Notably, patients with impaired renal function (eGFR ≤ 66 mL/min/1.73 m^2^) demonstrated significantly greater mortality reduction with preoperative β-blocker administration. These findings require prospective validation in targeted studies of this specific patient subgroup.

## Supplementary Material

supplementary materials.docx

## Data Availability

The data are not publicly available due to the No.717 Chinese Decree of the State Council, suggesting that the raw data of large samples cannot be shared globally. The code is available from the corresponding author upon request.
