# Supplementary material for: Heterogeneous effects of preoperative beta blockers on 30-day mortality after coronary artery bypass surgery
Source: Ann Med. 2026 Sep 4;58(1):2719256. doi: 10.1080/07853890.2026.2719256 (PMC13552291; doi:10.1080/07853890.2026.2719256)
Supplement: Supplementary file 1 [file IANN_A_2719256_SM5607.docx]

Table S1 Bootstrap validation of the associations between preoperative β-blocker therapy and 30-Day mortality across iCF-defined subgroups

| Subgroups | Unadjusted |  | Adjusted |  |
| --- | --- | --- | --- | --- |
|  | OR (95% CI) | *Significant percent（%）* | OR (95% CI) | *Significant percent（%）* |
| Subgroup 1 | 0.54 (0.25, 0.76) | 86.7 | 0.38 (0.20, 0.68) | 92.1 |
| Subgroup 2 | 2.63 (1.28, 7.17) | 70.4 | 2.29 (1.03, 6.56) | 47.4 |
| Subgroup 3 | 0.71 (0.38, 1.46) | 15.1 | 0.78 (0.38, 1.63) | 12.5 |
| Subgroup 4 | 0.83 (0.44, 2.30) | 2.20 | 0.82 (0.37, 2.20) | 5.70 |
| Subgroup 5 | 0.52 (0.09, 1.77) | 16.0 | 0.62 (0.08, 2.00) | 7.10 |

Note: Adjusted for symptomatic CAD, previous MI, previous PCI, CCS, hypertension, ACEI and age. The significant percent represents the proportion of bootstrap samples in which the association between preoperative β-blocker therapy and 30-day mortality reached statistical significance at a two-sided P < 0.05.

Table S2 Associations of 30-day all-cause mortality within iCF-defined subgroups after excluding patients with missing eGFR, LVEDD, or BMI data

| Subgroups | No. of events (%) | | Unadjusted |  | Adjusted |  |
| --- | --- | --- | --- | --- | --- | --- |
|  | No β-blockers | β-blockers | OR (95% CI) | *p* | OR (95% CI) | *p* |
| Subgroup 1 | 32/289 | 36/527 | 0.59 (0.36, 0.97) | 0.038 | 0.53 (0.31, 0.91) | 0.020 |
| Subgroup 2 | 7/290 | 23/491 | 1.99 (0.88, 5.06) | 0.117 | 1.68 (0.72, 4.43) | 0.256 |
| Subgroup 3 | 14/291 | 18/698 | 0.52 (0.26, 1.09) | 0.075 | 0.49 (0.23, 1.07) | 0.071 |
| Subgroup 4 | 14/337 | 18/660 | 0.65 (0.32, 1.34) | 0.230 | 0.75 (0.37, 1.59) | 0.449 |
| Subgroup 5 | 10/390 | 22/778 | 1.11 (0.53, 2.46) | 0.795 | 0.98 (0.46, 2.24) | 0.964 |

Note: Adjusted for symptomatic CAD, previous MI, previous PCI, CCS, hypertension, ACEI and age.

Table S3 Associations of 30-day all-cause mortality within iCF-defined subgroups after excluding patients with missing eGFR, LVEDD, or BMI data after additional adjustment for six major postoperative complications

| Subgroups | No. of events (%) | | Unadjusted |  | Adjusted |  |
| --- | --- | --- | --- | --- | --- | --- |
|  | No β-blockers | β-blockers | OR (95% CI) | *p* | OR (95% CI) | *p* |
| Subgroup 1 | 32/289 | 36/527 | 0.59 (0.36, 0.97) | 0.038 | 0.33 (0.17, 0.65) | 0.001 |
| Subgroup 2 | 7/290 | 23/491 | 1.99 (0.88, 5.06) | 0.117 | 1.73 (0.56, 6.25) | 0.362 |
| Subgroup 3 | 14/291 | 18/698 | 0.52 (0.26, 1.09) | 0.075 | 0.38 (0.13, 1.04) | 0.063 |
| Subgroup 4 | 14/337 | 18/660 | 0.65 (0.32, 1.34) | 0.230 | 0.59 (0.23, 1.46) | 0.249 |
| Subgroup 5 | 10/390 | 22/778 | 1.11 (0.53, 2.46) | 0.795 | 0.90 (0.32, 2.65) | 0.844 |

Note: Adjusted for symptomatic CAD, previous MI, previous PCI, CCS, hypertension, ACEI and age. Six available major postoperative complications were included in the outcome regression model: prolonged mechanical ventilation, reintubation, reoperation, postoperative myocardial infarction, postoperative stroke, and postoperative renal failure.

Table S4 Associations between preoperative β-blocker therapy and 30-Day mortality across iCF-defined subgroups: mixed-effects logistic regression models with hospital as a random intercept

| Subgroups | Unadjusted |  | Adjusted |  |
| --- | --- | --- | --- | --- |
|  | OR (95% CI) | *p* | OR (95% CI) | *p* |
| Subgroup 1 | 0.56 (0.33, 0.95) | 0.032 | 0.53 (0.30, 0.93) | 0.028 |
| Subgroup 2 | 1.55 (0.60, 4.04) | 0.372 | 1.34 (0.47, 3.83) | 0.581 |
| Subgroup 3 | 0.55 (0.24, 1.27) | 0.163 | 0.52 (0.22, 1.23) | 0.136 |
| Subgroup 4 | 0.63 (0.29, 1.38) | 0.247 | 0.78 (0.35, 1.72) | 0.536 |
| Subgroup 5 | 1.02 (0.45, 2.32) | 0.967 | 0.81 (0.33, 2.02) | 0.657 |

Note: Adjusted for symptomatic CAD, previous MI, previous PCI, CCS, hypertension, ACEI and age.

Figure S1: Study flow chart. LVEF: left ventricular ejection fraction; CABG, coronary artery bypass grafting; IABP, intra-aortic balloon pump, eGFR, estimated glomerular filtration rate; LVEDD, left ventricular end-diastolic diameter; BMI, body mass index; iCF, iterative causal forest.

Figure S2: Heatmap of Pearson correlation coefficients among candidate variables. Values are Pearson correlation coefficients; only correlations with an absolute coefficient >0.20 are labeled.
